# Health system lessons from the global fund-supported procurement and supply chain investments in Zimbabwe: a mixed methods study

**DOI:** 10.1186/s12913-024-11028-6

**Published:** 2024-05-01

**Authors:** Abaleng Lesego, Lawrence P. O. Were, Tsion Tsegaye, Rafiu Idris, Linden Morrison, Tatjana Peterson, Sheza Elhussein, Esther Antonio, Godfrey Magwindiri, Ivan Dumba, Cleyland Mtambirwa, Newman Madzikwa, Raiva Simbi, Misheck Ndlovu, Tom Achoki

**Affiliations:** 1 Africa Institute for Health Policy, P.O. Box 57266-00200, Nairobi, Kenya; 2 Department of Health Sciences & Department of Global Health, Boston University, Boston, U.S.A.; 3 Global Fund to Fight AIDs, Tuberculosis and Malaria, Geneva, Switzerland; 4 PricewaterhouseCoopers, Harare, Zimbabwe; 5 National Pharmaceutical Company of Zimbabwe, Harare, Zimbabwe; 6 Medicines Control Authority of Zimbabwe, Harare, Zimbabwe

**Keywords:** Health systems, Procurement and supply chain management, Global fund, Donor funded programs, Development assistance for health, Health systems strengthening

## Abstract

**Background:**

The Global Fund partnered with the Zimbabwean government to provide end-to-end support to strengthen the procurement and supply chain within the health system. This was accomplished through a series of strategic investments that included infrastructure and fleet improvement, training of personnel, modern equipment acquisition and warehouse optimisation. This assessment sought to determine the effects of the project on the health system.

**Methods:**

This study employed a mixed methods design combining quantitative and qualitative research methods. The quantitative part entailed a descriptive analysis of procurement and supply chain data from the Zimbabwe healthcare system covering 2018 – 2021. The qualitative part comprised key informant interviews using a structured interview guide. Informants included health system stakeholders privy to the Global Fund-supported initiatives in Zimbabwe. The data collected through the interviews were transcribed in full and subjected to thematic content analysis.

**Results:**

Approximately 90% of public health facilities were covered by the procurement and distribution system. Timeliness of order fulfillment (within 90 days) at the facility level improved from an average of 42% to over 90% within the 4-year implementation period. Stockout rates for HIV drugs and test kits declined by 14% and 49% respectively. Population coverage for HIV treatment for both adults and children remained consistently high despite the increasing prevalence of people living with HIV. The value of expired commodities was reduced by 93% over the 4-year period.

Majority of the system stakeholders interviewed agreed that support from Global Fund was instrumental in improving the country's procurement and supply chain capacity. Key areas include improved infrastructure and equipment, data and information systems, health workforce and financing. Many of the participants also cited the Global Fund-supported warehouse optimization as critical to improving inventory management practices.

**Conclusion:**

It is imperative for governments and donors keen to strengthen health systems to pay close attention to the procurement and distribution of medicines and health commodities. There is need to collaborate through joint planning and implementation to optimize the available resources. Organizational autonomy and sharing of best practices in management while strengthening accountability systems are fundamentally important in the efforts to build institutional capacity.

## Background

The Global Fund to Fight AIDS, Tuberculosis and Malaria (Global Fund), together with its Zimbabwean national and international stakeholders have continued to invest in health system strengthening to improve public health in the country. These investments have been guided by systemwide strategic assessments to understand the fundamental challenges facing the Zimbabwean health system [1–4].

Given the strategic importance of access to essential medicines, vaccines, and other health technologies as a strategic pillar of any health system [4, 5], the Global Fund supported the Ministry of Health and Child Care (MOHCC) through the United Nations Development Program (UNDP) to undertake a comprehensive assessment of the national procurement and supply chain management (PSCM) system in 2013 [6]. This assessment aimed to develop a strategic vision and costed action plan for improvement. This covered both upstream and downstream aspects of PSCM. The strategic purpose was to help the MOHCC launch a coordinated approach to invest in PSCM improvements and enhance coordination and cooperation in managing all health commodities across the health system [3, 6, 7].

Subsequently, the Global Fund supported several initiatives aimed at the realization of the improvements proposed by the comprehensive assessment undertaken by UNDP [6]. These initiatives primarily encompassed end-to-end supply chain assistance in critical areas, including demand quantification and forecasting, warehousing capacity development, fleet improvement, distribution systems, and waste management systems. Other support aspects focused on warehouse optimization, data and information management systems, and personnel training [7, 8]. In general, warehouse optimization is the process of improving the efficiency and effectiveness of warehouse operations. It involved refining workflows, leveraging technology, enhancing spatial utilization, and ensuring precise inventory management [6, 7].

More specifically, the Global Fund provided funds for the construction of warehouses for the National Pharmaceutical Company of Zimbabwe (NATPHARM) to facilitate the smooth handling of health commodities. Additionally, this support extended to water supplies (in terms of sinking boreholes) for various warehouses, water tanks, and booster pumps for the other branches. Global Fund also supported NATPHARM in constructing two incinerators in the two main cities, Harare, and Bulawayo to handle pharmaceutical waste effectively [9, 10].

Through Global Fund support, NATPHARM also received modern warehouse equipment such as forklifts, pallet jacks and rolling ladders, and data and information management system support for better visibility and effective handling of health commodities. The support further extended to optimising the Harare branch warehouse with modern receiving and transit capabilities to serve other feeder locations and the procurement of modern delivery trucks to facilitate the distribution of commodities. Global Fund support also extended to personnel training and retention for the effective functioning of NATPHARM and the broader procurement and supply system [3, 6, 9].

Focusing on quality assurance and safety of medicines and other health commodities, Global Fund supported the Medicines Control Authority of Zimbabwe (MCAZ) to develop capacity in quality testing of all commodities procured through grants. MCAZ was also supported with the installation of solar panels that allowed for an uninterrupted power supply to facilitate smooth operations at the organization. The Global Fund support was also critical in the upgrading of the biology and chemistry laboratories to attain WHO prequalification standards [8, 9, 11]. Similarly, there was direct support from the Global Fund to facilitate pharmacovigilance activities, such as adverse drug reactions reporting using electronic systems. All these measures were meant to ensure that the medicines and health commodities consumed in the Zimbabwean health system were safe and quality-assured [4, 5, 9, 11].

The overarching project by the Global Fund to support the Zimbabwean government was designed and implemented in response to the nationally identified gaps and opportunities that were established through various research and consultative efforts [3, 4, 6, 8]. The project was kickstarted in the first quarter of 2019 and continued through 2021, with various project components being implemented in a phased approach to achieve the national targets [3, 4, 7, 10, 11]. To ensure effective coordination, accountability, and avoidance of duplicative efforts, the project was designed and implemented in close coordination with other partners represented in the National Health Development Partners Coordination Forum (HDPCF), Health Sector Technical Working Group (HSTWG), and the Global Fund Country Coordination Mechanism (CCM), among others [3, 4, 6, 8, 10]. There was regular reporting to the respective coordination mechanisms to track progress as well as troubleshoot any implementation issues as they arose [6, 9].

Overall, the gaps identified through the various assessments commissioned by the Global Fund included a lack of effective coordination, poor inventory and order management, human resource constraints, and warehousing and storage inefficiencies [12, 13]. Therefore, the focus of the Global Fund support was to retool the Zimbabwean PSCM system to be efficient, cost-effective and responsive to the population's health needs, particularly in the face of global epidemics and pandemics such as HIV/AIDS and COVID-19 [14–18].

In an attempt to resolve these issues, healthcare systems around the world are working on streamlining their supply chains through various health system strengthening measures [19–22]. Therefore, the objective of this analysis was to assess the overall effects of the Global Fund-supported investments in the Zimbabwean PSCM system and document the lessons learned to inform future programming efforts to strengthen healthcare systems.

### Methods

**Fig. 1:**
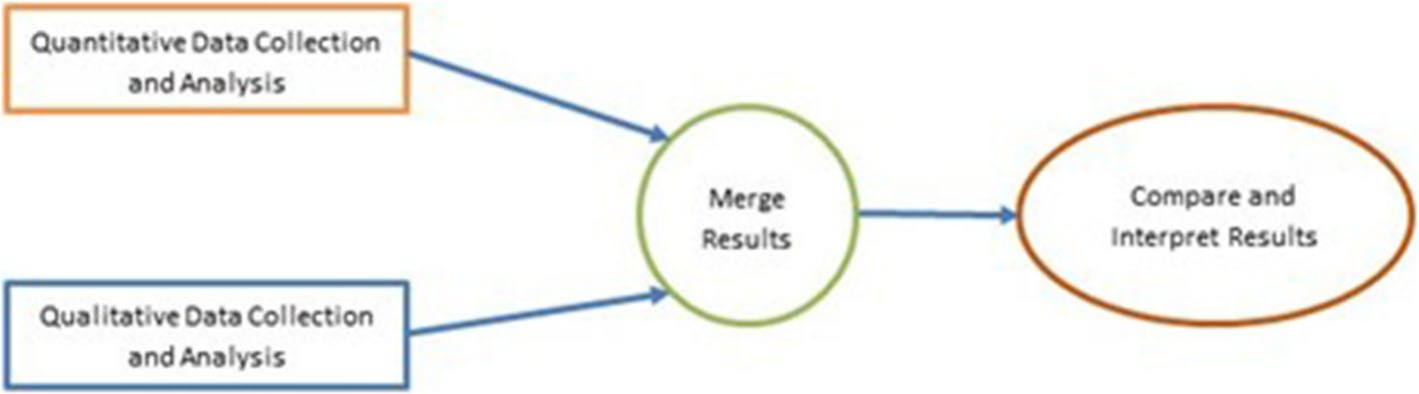
Convergent mixed methods design

The assessment covered a period of 2018 -2021 and employed both qualitative and quantitative research methods. Figure 1. illustrates the convergent mixed methods study design that was applied. In this study design, both the qualitative and quantitative data collection and analyses are implemented simultaneously, and the insights merged to provide a fuller picture [23].

The quantitative part of the study entailed collecting and analysing administrative data covering priority indicators that are routinely reported on the Zimbabwe PSCM. Meanwhile, the qualitative part comprised of key informant interviews (KII) focusing on stakeholders within the healthcare system to give perspective to the observed data trends. Insight from the two parts of the analysis were merged and subjected to comparative assessment and interpretation to ensure that a consistent picture emerged [23, 24]. More details on the methods are provided in later sections.

## Analytical framework

**Fig. 2:**
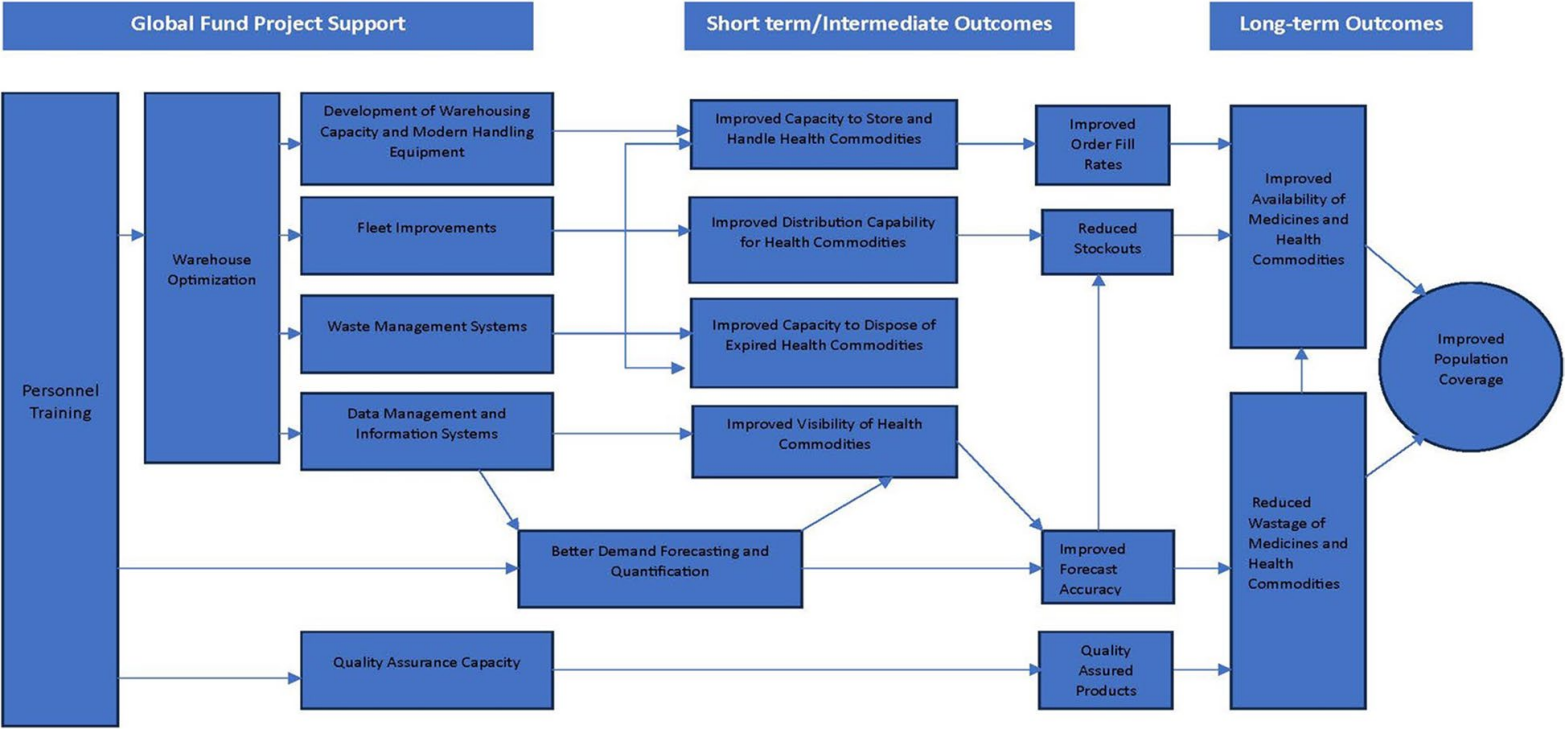
Analytic framework

The overall analytical approach espoused in this assessment was anchored on the logical relationships of the building blocks of the health system as described by the World Health Organization (WHO) health system framework [5]. Figure 2 shows the analytical framework, which illustrates the results chain cascading from the Global Fund-supported initiatives to the expected improvements in intermediate and long-term outcomes related to PSCM, including the availability of medicines, reduced wastage, and overall improvements in population-level coverage [4, 5].

Overall, the framework graphically displays the results Global Fund intended to achieve through its support to the Zimbabwean PSCM. The "theory of change" that underlies the Global Fund’s strategy is revealed through the arrows in the diagram that identify “causal” linkages through which various intermediate results interact to make progress toward the overall goal of improving health system performance [3, 5].

## Quantitative research

The quantitative research entailed a detailed descriptive analysis of the operational data that was routinely reported across the Zimbabwe PSCM system. Table 1. shows some of the key performance indicators (KPI) that were considered in our analysis.

**Table 1:** List of key performance indicators

Indicator	Relation to Analytical Framework
Population coverage	This indicator measures the ultimate performance of the system in terms of the proportion of the population in need that has access to the required medicine or health commodity
Order fill rates	This indicator assesses if the procurement and supply system meet the health system's needs on time
Stockout rates	This indicator is a measure of the capacity of the procurement and supply system to meet the needs of the health system with the priority health commodities
Commodity expiry rate	This indicator detects changes in wastage rates, e.g., due to expiries and other forms of wastage
Warehousing capacity	This indicator measures if the central warehouses have the space to hold the right products and quantities in demand
Forecast accuracy	This indicator determines if the procurement and supply system use data to guide its procurement decision making according to best practices
Stock variance	This indicator measures the difference between recorded quantity of stock items to what is physically available at a given point in time

## Data collection, management and analysis

The data used in this analysis were obtained from the routinely reported operational data that included the NATPHARM-operated warehouses and healthcare facilities in the country. The data were extracted from the various data management systems operated by the different institutions, cleaned, and collated into a comprehensive dataset in the form of a spreadsheet covering the period of the assessment. The database was examined for completeness and accuracy by cross-referencing the corresponding progress reports for specific periods. Trends of priority indicators were compared over time, as they related to Global Fund support to the PSCM space.

## Qualitative research

The qualitative assessment entailed KIIs with health system stakeholders who were knowledgeable and intimately involved in the Global Fund-supported initiatives and its intended beneficiaries. This included provincial and district management teams, hospital and clinic personnel, and other stakeholders in the Zimbabwe health system. Informed consent was obtained from each study participant involved in the study. The data collection protocol ensured that all study participants fully understood the objectives of the study and consented verbally to provide the required information.

As previously stated, the literature review helped map and identify critical organisations involved in the PSCM space, and more specifically, those involved in the procurement and health system strengthening activities supported by the Global Fund. A full list of those organisations is provided on Table 2.

**Table 2:** List of organization interviewed

Organization	Type of Organization	Number of Key Informants
Ministry of Health and Child Care	National Government	9
MCAZ	National Government	4
NATPHARM	National Government	5
United Nations Development Programme	Development Partner	5
World Health Organization	Development Partner	4
UNICEF	Development Partner	2
USAID	Development Partner	5
GHSC-PSM Project	Development Partner	2
Hospital/clinic	Non-governmental organization	4
Provincial and District Health Teams	Local Government	5
**Total**		45

## Sampling techniques

Convenience purposive sampling was used to select key informants and in-depth interviews [23]. Our sample was supplemented using snowball sampling methods (also called chain sampling). The initial respondents referred other potential respondents until no new information was forthcoming or achieved saturation. Efforts were made to be all-inclusive, involving various stakeholder groups and organisations intimately linked to the operations of the Zimbabwe PSCM landscape.

## Data collection, management and analysis

This comprised of KIIs using a structured interview guide that covered various thematic areas relevant to the assessment to obtain a comprehensive perspective of the impact of the Global Fund-supported initiatives in the country. In its development, testing and validation, the key informant guide was pretested and adapted to ensure suitability for the task. In view of the restrictions imposed to prevent the spread of COVID-19 infections at the time of the study, some KIIs were conducted online using multimedia channels such as Zoom, Skype, and telephonically.

Three research assistants supported the two project leaders in conducting the KIIs. After each interview, all notes taken by the research assistant were checked by the two project leaders to ensure completeness and readability to minimise recording errors. In addition, a tape recorder was used for interviews to assist with reference post data collection. All the recordings were stored in a pin-protected cloud storage which was only accessible by the two project evaluation leaders. Qualitative data obtained from the KIIs were transcribed in full and then manually analysed applying thematic content analysis. Where there was a divergence of opinion, an agreement was established through discussion with three members of the project evaluation team. In thematic analysis, data from interview transcripts were grouped into similar concepts. This approach was appropriate for semi-structured expert interviews as it is used to code text with a predefined coding system that can then be refined and completed with new themes emerging [23, 24]. Our initial coding system was defined during the desk review stage and continuously updated in the successive phases of data collection employing a deductive approach of qualitative research. The emerging themes were not preconceived (desk review) but emerged from the data during the coding process, while the global themes were the highest-order themes that emerged from the data and were broad enough to capture the essence of the entire dataset [23]. The codes are presented in a tabular format in the results section below.

## Results

This section presents both the quantitative and qualitative research results from the study. The quantitative results comprise of trends of the priority operational PSCM indicators for the relevant period. The qualitative results present the perspectives of the key health stakeholders involved in the Zimbabwe healthcare system.

## Quantitative results

Table 3 shows that the total warehouse capacity across the Zimbabwean health system increased by 37.8% between 2018 and 2021.

**Table 3:** Priority performance indicators

Year	National Target	2018	2019	2020	2021	Percentage Change
Warehouse Space (M^2^)	-	21,050	21,050	27,000	29,000	37.8%
Health Facility Coverage	100	88	91	96	100	13.6%
Order fulfillment within 90Days	100	39	40	48	91	133.1%
Order Fill Rate (TLE 600mg)	100	53.1	67.7	33.5	72.5	36.5%
Stock Out Rate (TLE 600mg)	0	11.6	17.2	8.8	10.0	-14.5%
Order Fill Rate (Determine HIV Test Kit)	100	79.1	64.0	44.7	82.7	5%
Stock Out Rate (Determine HIV Test Kit)	0	9.4	16.5	6.8	4.8	-49%

Of the 1500 public health facilities in Zimbabwe coverage by the PSCM system was consistently high between the years 2018 and 2021, averaging 94%, and increasing by 13.6% over the same period. However, order fulfillment rate within 90 days, for 1410 reporting health facilities was consistently below 50% from 2018 to 2020, despite the reported high coverage for the health facilities by the PSCM in the country. Notably, this indicator showed remarkable improvement to 91% in 2021, from an average of 42% from the previous three years. More specifically, the order fill rate for Tenofovir 300mg/ lamivudine 300mg/efavirenz 600mg (TLE 600mg) improved despite the significant drop observed in 2020. However, when comparing 2018 and 2021, the order fill rate for this specific HIV drug increased by around 36.5%, while the stockout rates for the same drug at the central stores declined by about 14.5% over the same period.

Table 3 further shows a 44% drop of order fill rates for the Determine HIV Test Kit between 2018 and 2020 for the 1410 reporting health facilities, only to recover in the year 2021, where order fill rates improved to 83%. At the same time, the stockout rates for the Determine HIV Test Kit at the central stores declined by 49% between 2018 and 2021.

**Fig. 3:**
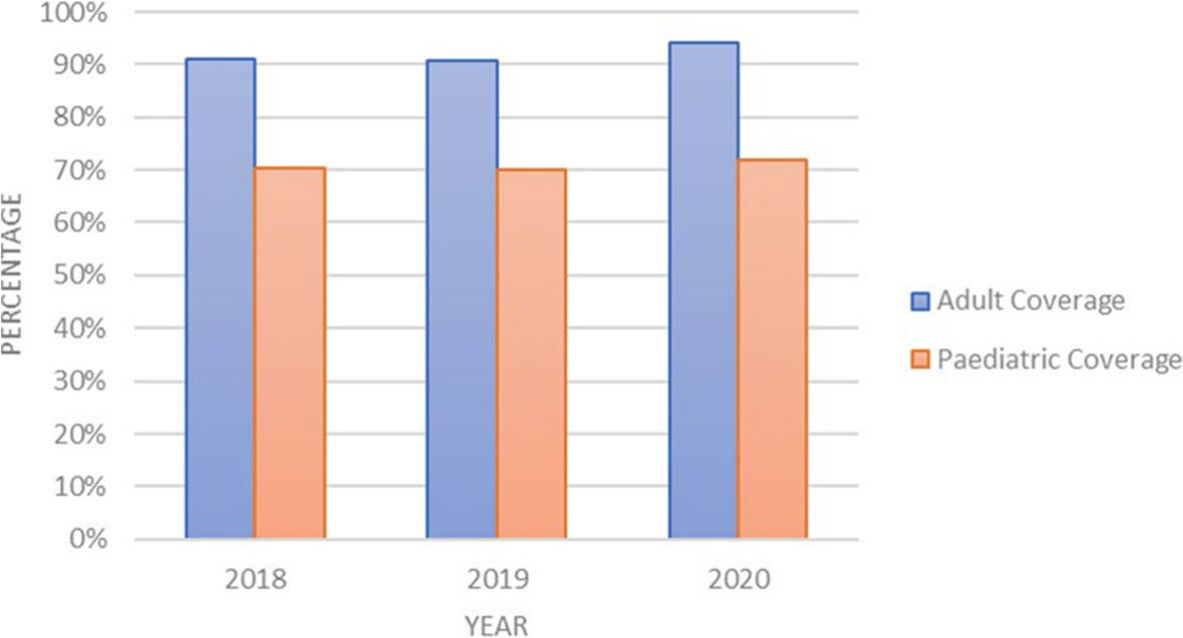
Adult and paediatric HIV treatment population coverage

Figure 3 shows the estimated average population coverage for HIV treatment for adults and children, from 2018 to 2020, at 92% and 71%, respectively. The figure shows that there was limited variation in the population level coverage over the years, despite the estimated increase in the number of people living with HIV over the same period. The national target for this indicator is 95%.

**Fig. 4:**
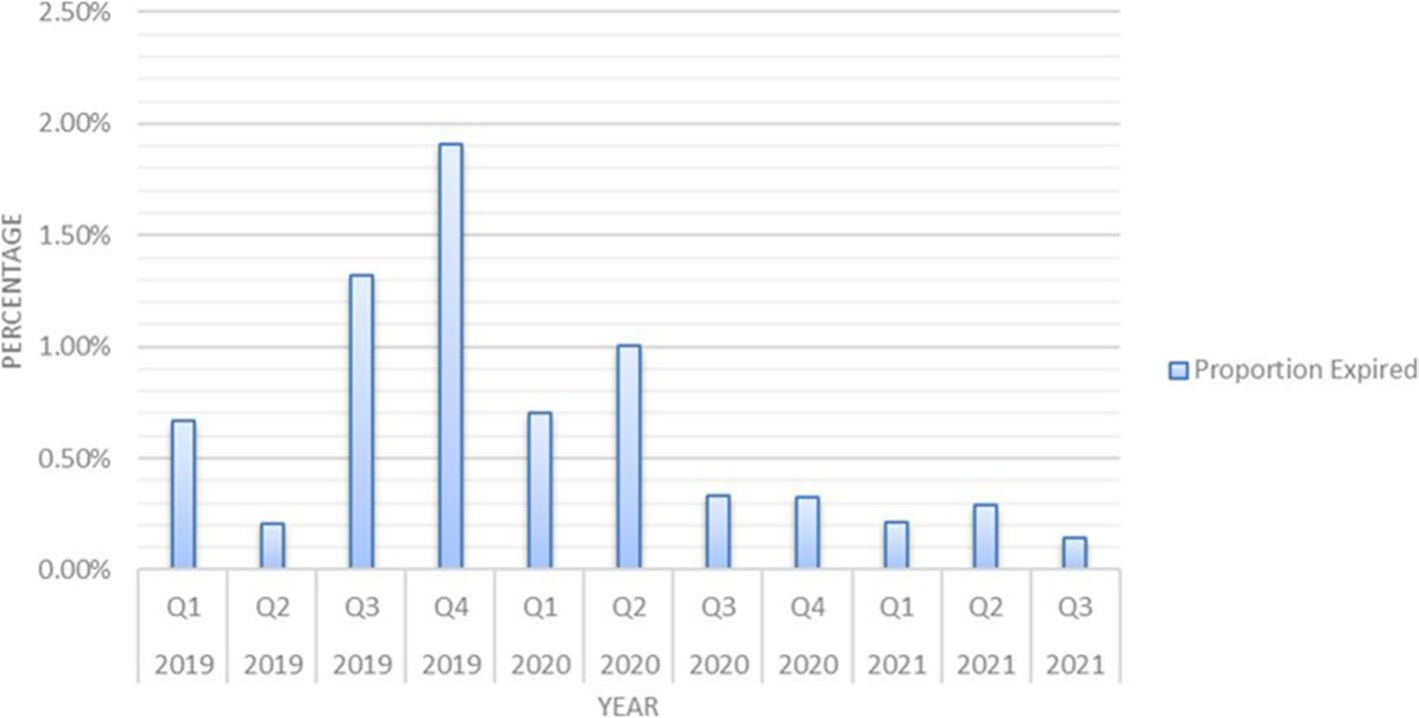
Percentage value of expired stock

Figure 4 shows the proportion of the value of the expired stock in the 7 warehouses, over three years, between 2019 and 2021, which demonstrates a declining trend over time. The highest expiry was in quarter 4 2019 at 1.9%, compared with the lowest in quarter 3 2021 at 0.1%. This represents a 93% reduction in value of expired stock.

**Fig. 5:**
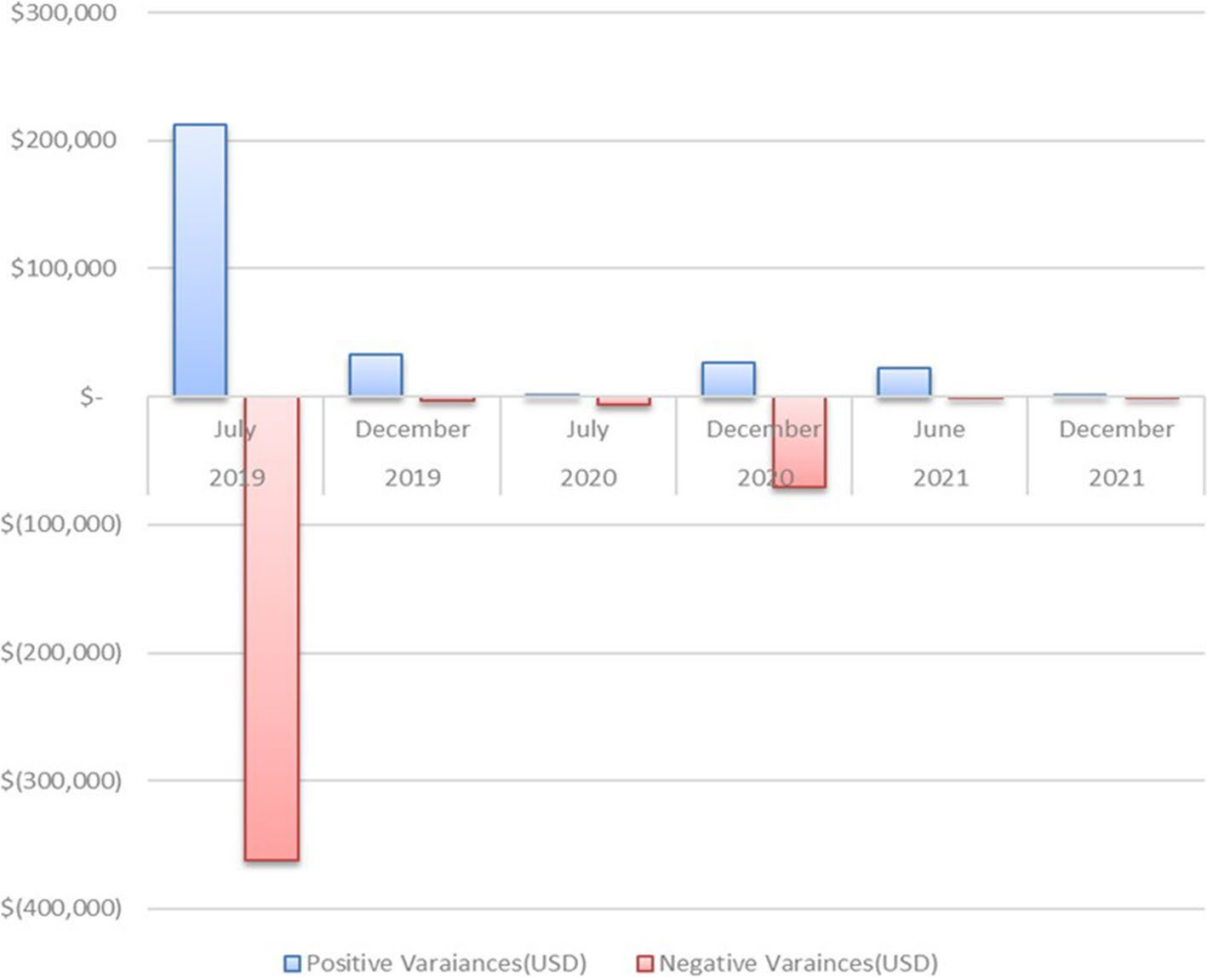
Stock-taking variance

Figure 5 shows the combined stock-taking variance valued in United States Dollar terms across 7 warehouses over a three-year period. The stock variance shows a declining trend over the three-year period to negligible values at the end of 2021.

**Fig. 6:**
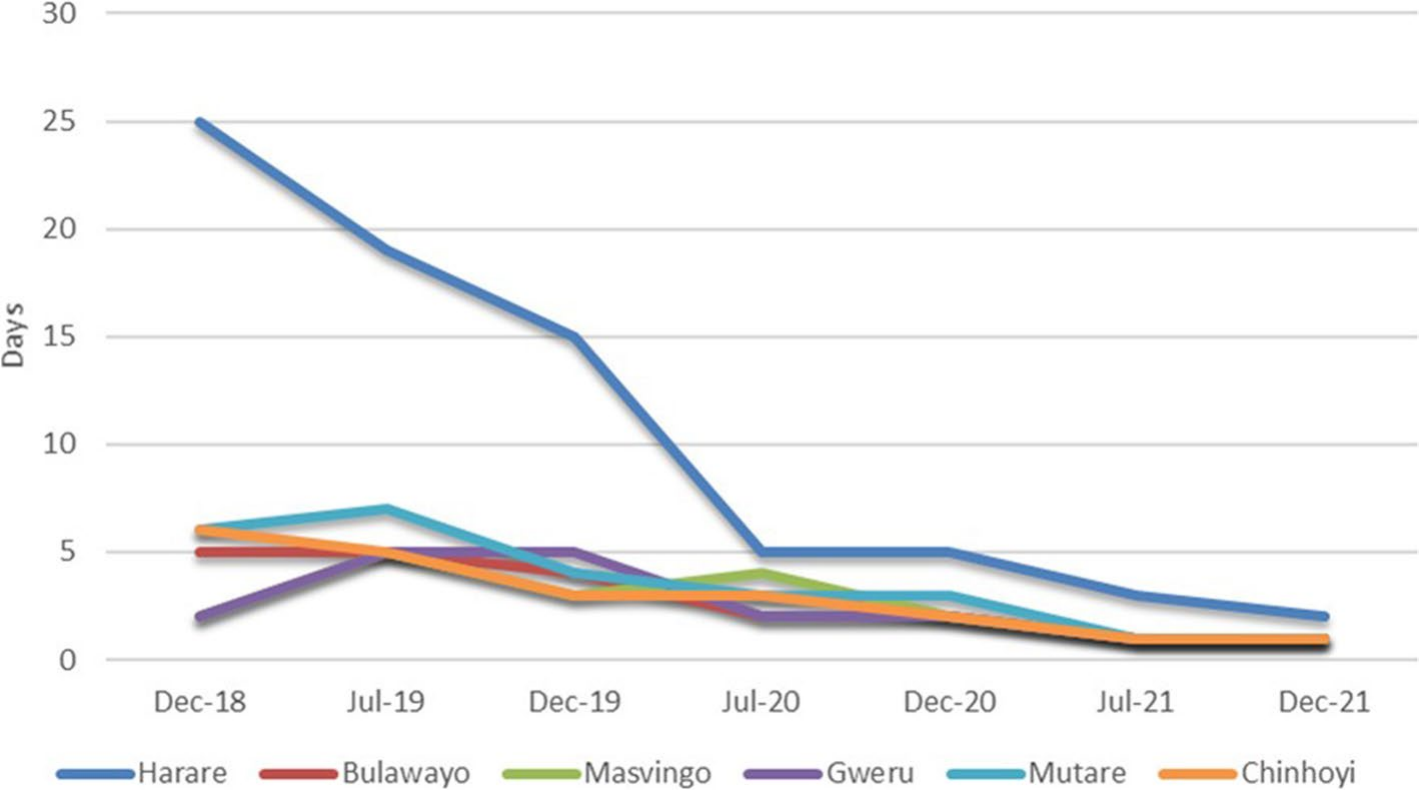
Duration to resolve stock variance

Figure 6 shows the temporal trend of the number of days that it took NATPHARM to resolve the stock variances across the different warehouses in the country. Overall, there is a decline from the average of 8 days from the December 2018 stocktake (with Harare warehouse as an outliner at 25 days), to an average of 1 day in the December 2021 stocktake, where all warehouses converge.

Table 4 shows the funding levels in USD$ to support the diagnostic capacity for Covid-19, comprising of the polymerase chain reaction test (PCR) and rapid diagnostic tests (RDT). The table further shows the PSCM related costs, the total test done, and positive cases identified over the two-year period. The total funding between 2020 and 2021 increased by 290%, with testing levels increasing by 490% over the same period. The average Covid-19 positivity rate in 2020 was 6.4% while the positivity rate for 2021, was 3.1%, indicating a greater than 50% drop.

**Table 4:** Covid-19 commodities and testing capacity

Item Description	Units	Year 2020	Year 2021	Percentage Change
PCR and Consumables	US$	2,498,016	4,964,589	98.7%
RDTs	US$	-	4,243,509	-
PSCM Costs	US$	524,583	2,578,267	391.5%
Total Funding	US$	3,022,599	11,786,365	289.9%
Covid Tests Done	Number	216,254	1,274,724	489.5%
Covid Cases Confirmed	Number	13,867	40,080	189.0%
Positivity Rate		6.4%	3.1%	-51.0%

## Qualitative results

Most of the participants interviewed acknowledged that the Global Fund support to NATPHARM and the broader Zimbabwean health system had been central in improving the overall performance of the health system through improved availability of essential medicines and other health commodities. This was largely achieved by ramping up the various components of the PSCM value chain and related operations, leading to efficiency, effectiveness and reliability.

Table 5 shows the codes, emerging and global themes from the thematic content analysis. The emerging themes revolved around the lack of infrastructure and equipment curtailing warehouse operations before the Global Fund support. Data gaps and poor product visibility were also emerging themes, as were the effects of the old fleet on the overall supply and distribution system. Similarly, issues of infrastructure, capacity, and personnel training gaps emerged as crucial themes hindering quality assurance within the PSCM. Global themes also largely focused on infrastructural inadequacy leading to underperformance. Improvements leading to better handling of commodities; data and information systems, enhancing visibility and supporting accuracy in forecasts; improvements in the distribution systems enabled by newer fleets also featured as global themes. Similarly, better trained and motivated personnel, able to perform critical functions; capacity to ensure the quality and safety of medicines and other health commodities; and the need for effective multistakeholder partnerships to improve effectiveness and sustainability of health systems, were key themes.

**Table 5:** Codes and emerging themes

Code	Emerging Themes	Global Themes
Limited storage capacity hindered the NATPHARM operations and led to inefficiencies	Limited infrastructure and equipment curtailed operations	Infrastructural inadequacy led to underperformance
Handling of medical commodities was difficult in old warehouses		
We did not have the proper equipment for handling and storing commodities	Poor handling and visibility of medicines and health commodities in storage led to wastage and inefficiencies	The inefficient value chain that needed to be optimised
We had limited visibility of the commodities at hand, leading to wastage and expiries		
The expired drugs occupied a lot of essential space		
The MCAZ has the capacity to perform all the necessary safety and quality assurance tests	Improved capacity to perform safety and quality assurance for health commodities	Quality assurance and safety are critical functions for effective procurement functions
The MCAZ is able to support quality assurance tests for the national and regional procurement activities		
Without good data on demand for specific commodities, we often overstocked, leading to wastage across the health system	Data gaps resulted in operational challenges e.g., overstocking and wastage	Improved reporting and data use lead to better decisions in health commodity management
Better visibility of medicines and health commodities across the system now allows for better demand forecast		
Reporting has improved with the training and better information management systems	Improved data use due to personnel training	
The old Fleet was costly to run, and the newer vehicles allow for faster and timely delivery	Ineffective and inefficient distribution systems	Improved fleet for better delivery
Stockouts used to be the order to the day		
With the training, we can forecast demand better	Training improved capacity to perform	Trained and motivated personnel for better management
The staff are motivated and better able to handle the various functions		
Government and donors need to work together to improve the health system	Working together for sustainability	Multistakeholder partnership for sustainability
Building local capacity is essential for the sustainability of health systems		

## NATPHARM operations

According to the NATPHARM management, warehouse improvement and optimisation exercise resulted in better visibility and improved efficiency in the operations related to the commodity handling across the entire value chain. More specifically, the processes related to stock taking improved markedly over time according to the reports presented by various organizations that had been commissioned to undertake the stock audits.

“*…. warehouse optimisation supported implementing an inventory management system which conformed with bin location and variant codes, according to different donors. The result was improved, faster and more accurate stock takes, a sharp reduction of variances and more streamlined order processing*” Participant, NATPHARM.

Further, it was reported by various participants that order processing and deliveries had improved to be timely and on schedule as a result of the improved visibility and efficiency harnessed across the PSCM. Similarly, there was consensus that receiving processes and documentation had significantly improved through the support offered by Global Fund particularly towards warehouse optimization. The improvements in the data management systems and related trainings were also cited as contributory to the overall trend that was observed.

“*Reporting quality has greatly improved and is now timely, accurate and complete. This helps in accurate forecasting of demand, which in turn avoids unnecessary wastage and expiries”* Participant MOHCC.

There was consensus from the majority of participants interviewed that the fleet improvements had improved the availability of essential commodities vital for the effective management of high burden diseases; HIV/AIDS, Malaria and Tuberculosis in Zimbabwe. According to participants from a local health facility, this was evidenced by low stockout rates for the key commodities needed to manage these three conditions effectively. The new fleet was reported to facilitate deliveries from various warehouses to the recipient health facilities on a regular basis. This level of distributional access coupled with better demand forecasting as a result of improved data use, was noted as critical in the improved availability of medicines and health commodities at the health facility levels.

Further, respondents in the leadership of NATPHARM revealed that the Global Fund support had benefited the overall financial position of the organisation by tapping into efficiencies harnessed through the various measures that have been implemented. Some of the support measures that resulted in efficiency improvements include, the warehouse optimization, pharmaceutical waste management and fleet improvements, which ultimately reduced operational costs.

For example, it was noted that running a newer fleet of vehicles led to lower maintenance and fuelling costs than previously was the case, when deliveries were done using older vehicles. Similarly, it was noted that pharmaceutical waste resulting from expired medicines and other health commodities was expensive to store and dispose, particularly when engaging third party organizations. However, this additional cost was reportedly in the decline, as a result of the investment in the incinerators for waste management.

“*The provision of incinerators for waste management has resulted in huge savings in terms of the cost of waste destruction. It has also resulted in significant compliance with environmental health regulations.”,* Participant, NATPHARM.

## MCAZ operations

Majority of the participants agreed that the Global Fund support to MCAZ strengthened its overall capacity to handle the requisite safety and quality assurance needs to effectively support the procurement functions for medicines and other health commodities within the country and regionally. The installation of solar panels to provide uninterrupted electricity power supply for the operations of the organization was cited as a huge advantage allowing for improved performance, in a country where power supply is unreliable. Similarly, other participants cited, the support for MCAZ laboratories to obtain the WHO prequalification status, as a major step towards effectiveness and sustainability for the organization; citing the fact that MCAZ is offering quality assurance services regionally at a fee.

“*We [MCAZ] now have the capacity to conduct the safety and quality assurance tests needed to support the procurement of commodities in the country and the region. We [MCAZ] even recently won the tender to support the regional procurement activities*”, Participant, MCAZ.

## Discussion

Based on the results framework provided in Fig. 3, there is clear evidence that the Global Fund-supported initiatives resulted in positive improvements in the overall performance of the Zimbabwean PSCM system. However, it is important to recognise some of the assessment’s limitations in interpreting these findings. First, the results reported are for a limited observation period and a limited set of indicators, which are largely confined to the national level analysis, missing out on granular subnational and commodity-specific analysis that could be more informative. Secondly, this study was not conceptualised before the onset of the intervention reported here (i.e., Global Fund-supported initiatives), and therefore, no specific steps were taken to develop an appropriate prospective research design and data collection strategy to support a more rigorous assessment. Therefore, the study relied on secondary PSCM data that were sparse and covered a limited period. Third, the study could be subject to confounding relationships with other concurrent interventions being implemented by other health system stakeholders that have direct or indirect effects on the PSCM system, complicating impact attribution to specific interventions. Forth, the analysis focused only on a narrow subset of medicines and commodities related to HIV/AIDS and COVID-19. However, despite these limitations, every effort has been made to use the most up-to-date and complete information available, including validation using official reports and collaborative reported data with key informant interviews.

The estimated population coverage for HIV treatment for both adults and children remained consistently high despite the increasing prevalence in the country. It was estimated that adults living with HIV increased by 10% from a baseline of 2018, to reach 1.3M in 2020, while children living with HIV increased by 24%, from a baseline of 2018, to reach 75 000 in 2020 [3, 4, 9]. As a key last mile population outcome, it can be rightly assumed that high HIV treatment coverage in the Zimbabwean system emanated from strengthened inventory management functionality and improved delivery of orders supported by a modern fleet of vehicles, which allowed for meeting the supply target of four quarterly rounds [3, 8]. Population coverage is an important performance measure for a health system. It unites two important concepts; need and utilisation of an intervention to improve health [25]. In our case, the intervention is HIV treatment and the population in need is those living with HIV needing treatment; and the proportion with access and able to use the treatment they need, represents population coverage. This is a fundamentally important consideration as various health systems, including low- and middle-income countries, are making universal health coverage (UHC) efforts. There is no question, that improved access to essential medicines and other health technologies is a fundamental cornerstone towards UHC [1, 5, 26].

Other intermediate indicators that are critical for progress towards improved availability of medicines and other health commodities and hence UHC, also showed significant improvements that could be attributed to Global Fund-supported initiatives. For example, reduced wastage and decreasing value of expired health commodities reported, point towards improving efficiency across the value chain. As noted earlier, efficiency is one of the fundamental expectations of an effective health system outlined in the WHO health system framework [3, 5]. The diminishing value of expiries could be ascribed to various factors, including the improved workflow processes and data accuracy at NATPHARM. This improvement which is associated with better visibility of commodities across the value chain could be attributed to investments made by Global Fund such as the enterprise resource planning platform, coupled with concomitant training and supervision.

Through Global Fund’s assistance to NATPHARM, automation of tasks such as stock management, ordering, and other operational activities was central and contributory to driving the observed improvements in the handling of commodities; reduction of wastage and expiries and improving availability. Similarly, better inventory management and warehouse optimization activities such as decongestion resulted in quicker, timely, more accurate, and well-documented stock takes, improving overall commodity management.

Variances between stock on hand and physical counts were used to determine whether facilities are conducting period checks on their stocks and therefore calculating monthly consumption of commodities accurately. As such the variance across commodities should be zero. Low variance indicates that the stocks at hand are generally similar and do not vary widely from the physical stock counts, while high variance indicates that the respective values have greater variability and are more widely dispersed from one another. There is clear evidence pointing towards the reduction in stock variances when comparing stock on hand and physical counts across the different warehouses in the country over time. This trend can be attributed to better visibility of commodities at the warehouses and training of personnel which was supported by the Global Fund [2, 9]. Similarly, the number of days it took the NATPHARM personnel to resolve stock variances showed a dramatic reduction, from an average of 8 days to 1 day in a span of 3 years. This observed trend could also further support the claim that overall, the Global Fund supported initiatives produced the desired results.

With the advent of Covid-19, the effects of the Global Fund support on the PSCM became evident considering the robust response the country was able to mount particularly in terms of diagnostics [9]. The country was able to rapidly roll out COVID-19 testing, reaching many people between 2020 and 2021. Similarly, the Covid-19 positivity rates declined from 6.4% to about 3.1% over the same period. High positivity rates may indicate that the health system is only testing the sickest patients who seek medical attention and is not casting a wide enough net to know how much of the virus is spreading within its communities. A low rate of positivity on the other hand, can be seen as a sign that a health system has sufficient testing capacity for the size of the Covid-19 outbreak and is testing enough of its population to make informed decisions about reopening the economy. The WHO guidance is that countries which have conducted extensive testing for COVID-19, should remain at 5% or lower positivity rate for at least 14 days.

Safe pharmaceutical waste management and disposal is a primary consideration of any effective health system in completing the PSCM loop [27]. The Global Fund supported the investment in MOHCC operated incinerators. These investments could largely be associated with reduction in the cost of storage, handling and disposal of the expired stock, particularly when considering that certain space was rented from third parties which often charged a premium. Safe pharmaceutical waste disposal also became more priority with the increased supplies that resulted from the efforts to tackle the Covid-19 pandemic.

Despite signs of progress, there was temporary faltering of indicators- namely, order fill and stockout rates; associated with key commodities for effective management of HIV in the year 2020, warranting an explanation. The drop in Tenofovir/Lamivudine/Efavirenz (TLE 600mg) in 2020 could be linked to several factors. In the year 2019, the Zimbabwe MOHCC adopted new treatment regimens containing Dolutegravir. This means, newly HIV positive clients were started on Dolutegravir regimen as standard of care rather than the previous first line treatment which then surged Tenofovir/Lamivudine/Dolutegravir 50mg order fill rate, while having the opposite effect on the old regimen. Lastly, the effects of COVID-19 pandemic cannot be underestimated as the global supply chain systems were logged with delays which caused disruptions and inefficiencies in health systems in many countries [28]. In the same period, Determine HIV Test Kit rebounded from stocking out in central stores because of strengthened warehouse optimization activities, including better inventory management, purposeful stock taking, and approval processes contributed to the lowering of stockout rates.

The Global Fund-supported initiatives were also instrumental in building capacity by training key personnel for the effective implementation of activities related to the procurement and supply chain management function [8, 29, 30]. Better quantification and forecasting capabilities (due to data availability through e-LMIS and personnel training), improved warehousing capacity to hold a wide portfolio of products, and direct delivery to facilities through a modern fleet could have contributed to the high population coverage reported [26, 30, 31]. According to the WHO health system framework, effective leadership is required to coordinate all the functions of the health system in order to achieve the desired outcomes [5]. Therefore, it is sensible to conclude that, the reported health system improvements could not have happened without effective leadership and well-trained staff tasked with coordination and management across the PSCM value chain. It can be further inferred that the training and capacity development measures offered to the NATPHARM personnel were consequential in supporting the broader health system to meet its overall objectives, including improving PSCM performance [2, 7, 29].

Similarly, adequate infrastructure, equipment, data, and information management systems are crucial ingredients for a well-functioning health system, according to the WHO health system framework [4, 5, 9]. The Global Fund-supported initiatives were central in supporting these aspects of the health system through improved warehousing capacity, of modern equipment, installation of solar panels, fleet improvement and deployment of an electronic-logistic management information system (e-LMIS). The cumulative benefits of these investments include optimised procurement and distribution of commodities leading to a reduction in stockout rates and timely order refills to meet the population health needs [9].

Based on these findings, it would be reasonable to conclude that the Global Fund-supported initiatives in Zimbabwe contributed positively to strengthening the health system, particularly through the improved performance of the various indicators linked to the PSCM system at national and regional warehouses, as well as health facilities. Considering the prevailing health needs in the country, the implementation of this project and the manner of investments provide a basis and playbook for further support to make progress. This is particularly true considering the various competing priorities in the Zimbabwean healthcare system amidst resource constraints [3, 6, 8]. This was largely underpinned on the overarching focus on UHC and the critical role that an effective PSCM plays towards that very objective [3, 12–14].

The Global Fund-supported project in Zimbabwe worked through the existing national coordination mechanisms where various key stakeholders, including MOHCC and NATPHARM, were involved in all key strategic planning and implementation decisions, ensuring country leadership and ownership. It was clear from the outset that this approach required sound partnership, transparency, and accountability among all the involved stakeholders, to deliberate and find common ground, guided by the overarching objective to make progress towards UHC.

## Conclusion

The question of securing the gains and ensuring sustainability is fundamental for donor supported health programs in low- and middle- income countries. To make progress, it is imperative for health system stakeholders, including governments and donor organizations that are keen to sustainably strengthen health systems to pay close attention to critical areas like the procurement and distribution of health commodities. It is critical to collaborate with key stakeholders through joint planning and implementation to optimize the available resources. Organizational autonomy coupled with strong data driven accountability systems and the sharing of best management practices are fundamentally important in this discourse.

## Data Availability

The datasets used and/or analysed during the current study are available from the corresponding author on reasonable request and once written permission is obtained from NATPHARM.

## Abbreviations

CCM: Country Coordination Mechanism
HDPCF: Health Development Partners Coordination Forum
HSTWG: Health Sector Technical Working Group
KII: Key Informant Interview
MCAZ: Medicines Control Authority of Zimbabwe
MOHCC: Ministry of Health and Child Care
NATPHARM: National Pharmaceutical Company of Zimbabwe
PCR: Polymerase Chain Reaction
PSCM: Procurement and Supply Chain Management
RDT: Rapid Diagnostic Tests
TLE: Tenofovir Lamivudine Efavirenz
UHC: Universal Health Coverage
UNDP: United Nations Development Program
WHO: World Health Organization
